# Timely Recognition and Early Multi-Step Antinflammatory Therapy May Prevent ICU Admission of Patients With MIS-C: Proposal for a Severity Score

**DOI:** 10.3389/fped.2021.783745

**Published:** 2021-12-20

**Authors:** Giacomo Brisca, Alessandro Consolaro, Roberta Caorsi, Daniela Pirlo, Giulia Tuo, Claudia Campanello, Elio Castagnola, Andrea Moscatelli, Marco Gattorno, Angelo Ravelli

**Affiliations:** ^1^Terapia Semintensiva, IRCCS Istituto Giannina Gaslini, Genoa, Italy; ^2^Clinica Pediatrica e Reumatologia, IRCCS Istituto Giannina Gaslini, Genoa, Italy; ^3^Dipartimento di Neuroscienze, Riabilitazione, Oftalmologia, Genetica e Scienze Materno-Infantili (DiNOGMI), Università Degli Studi di Genova, Genoa, Italy; ^4^UOC Cardiologia, IRCCS Istituto Giannina Gaslini, Genoa, Italy; ^5^UOC Malattie Infettive, IRCCS Istituto Giannina Gaslini, Genoa, Italy; ^6^Direzione Scientifica, IRCCS Istituto Giannina Gaslini, Genoa, Italy

**Keywords:** anakinra, multi-step anti-inflammatory treatment, SARS-CoV-2, pediatric COVID-19, immunoglobulins, kawasaki disease, multisystem inflammatory syndrome in children, intensive care unit

## Abstract

In this observational study, we report the clinical, therapeutics and outcome features of 23 patients with multisystem inflammatory syndrome (MIS-C) who have been treated in Gaslini Children Hospital (Genoa, Italy) with a multistep antinflammatory treatment protocol, based on disease severity at admission. Patients were initially assigned to four severity classes on admission and treated accordingly. The therapeutic options ranged from IV immunoglobulin alone to a combination of IVIG plus pulses of methylprednisolone plus anakinra for patients with marked cardiac function impairment or signs of macrophage activation syndrome, with rapid treatment escalation in case of inadequate therapeutic response. With the application of this therapeutic strategy, no patient required admission to Intensive Care Unit (ICU) or invasive mechanical ventilation, and no inotropic drugs administration was required. Early aggressive treatment of MIS-C, with therapeutic interventions modulated based on the severity of clinical manifestations may help to prevent the progression of the inflammatory process and to avoid the need of admission to the ICU. A timely intervention with anti-IL-1 blockers can play a pivotal role in very severe patients that are at risk to have an incomplete response to immunoglobulins and steroids.

## Introduction

In the spring of 2020, a multisystem inflammatory syndrome in children (MIS-C) emerged in countries mostly hit by COVID-19 (1–4). Strong viral and epidemiological evidence suggested that SARS-CoV-2 was the trigger of the syndrome. However, the observation of a time lag of 2–6 weeks between the peak of SARS-CoV-2 infection and the onset of MIS-C indicated that the virus acted as a trigger of a post-infectious inflammatory process. (5).

All patients with MIS-C present with fever, and a sizeable proportion of them display some of the typical manifestations of Kawasaki disease (KD), especially rash, cheilitis, and bilateral non-secretive conjunctival injection. However, most cases develop symptoms that are uncommonly seen in KD, such as gastrointestinal complaints (abdominal pain, vomiting and diarrhea), myocarditis associated with cardiogenic-vasoplegic shock, and neurological abnormalities (meningitis-like symptoms or encephalitis) (6). Impaired cardiac function and shock necessitate admission to the Intensive Care Unit (ICU) for 60–80% of patients, and half of them require inotropes and/or fluid resuscitation (5, 7). The fatality rate is approximately 2% (8, 9).

Most patients with MIS-C have been treated with intravenous immunoglobulin (IVIG), alone or in combination with glucocorticoids (10–12). In severe or refractory cases, particularly when myocarditis was present, biologic response modifiers, particularly tumor necrosis factor or interleukin-1 inhibitors, were given (13, 14). However, despite the publication of various therapeutic recommendations (15–17) the treatment of MIS-C remains empirical and little information is available about the effectiveness of proposed strategies.

We describe herein our experience with a therapeutic protocol based on an early aggressive multi-step therapy, which enabled quick control of the inflammatory process and avoided admission to the ICU of all patients with MIS-C seen at our hospital.

## Methods

All consecutive patients seen at the Gaslini Institute of Genoa, Italy from 1^st^ April 2020 to 1^st^ June 2021 who met the case definition of MIS-C (18, 19) were included in the study.

This study was reviewed and approved by the Regione Liguria Ethical Board (IRB# 370/2020). Written informed consent to participate in this study was provided by the participants' legal guardian/next of kin.

All children were treated according to an internal multistep treatment protocol originally devised for KD by expert pediatric rheumatologists (AC, RC, MG, AR) and infectious disease specialists (EC).

Patients were initially assigned to four severity classes developed in our Institute on the basis of their clinical features on admission, the severity of cardiac involvement expressed by echocardiographic findings or cardiac enzymes levels and/or the occurrence of blood tests abnormalities suggestive for macrophage activation syndrome (Table 1). The severity classes' descriptors were progressively adapted to the evolving scenario and the growing body of evidence on the new clinical entity.

**Table 1:** The “Gaslini severity assessment tool” for MIS-C.

**Severity classes**	**MIS-C case definition**	**Complete-incomplete KD criteria**	Severe abdominal involvement^*^	**Signs of cardiac dysfunction at echocardiography**	**Increased cardiac enzymes**	**Laboratory features of MAS**
Class I	Yes	Yes	No	No	No	No
Class II	Yes	Yes/No	No	Cardiac dyskinesia with normal ejection fraction	No	No
Class III	Yes	Yes/No	Yes	Cardiac dysfunction with ejection fraction <50% and > 35%	Increased troponin and/or NT-pro BNP > 1,000 pg/ml	Increased ferritin (<1,000 ng/ml)
Class IV	Yes	Yes/No	Yes/No	Cardiac dysfunction with ejection fraction <35% and/or hypotension/shock		Increased ferritin (> 1,000 ng/ml and/or cytopenia)

*MIS-C, multisystem inflammatory syndrome in children; KD, Kawasaki disease; MAS, macrophage activation syndrome; NT- pro BNP, N-terminal B-type natriuretic peptide level.*

* *Severe abdominal involvement was defined based on the presence of persisting severe abdominal pain, persisting vomiting and/or diarrhea, acute abdomen signs, ascites, pseudo-appendicitis*.

**Figure 1:**
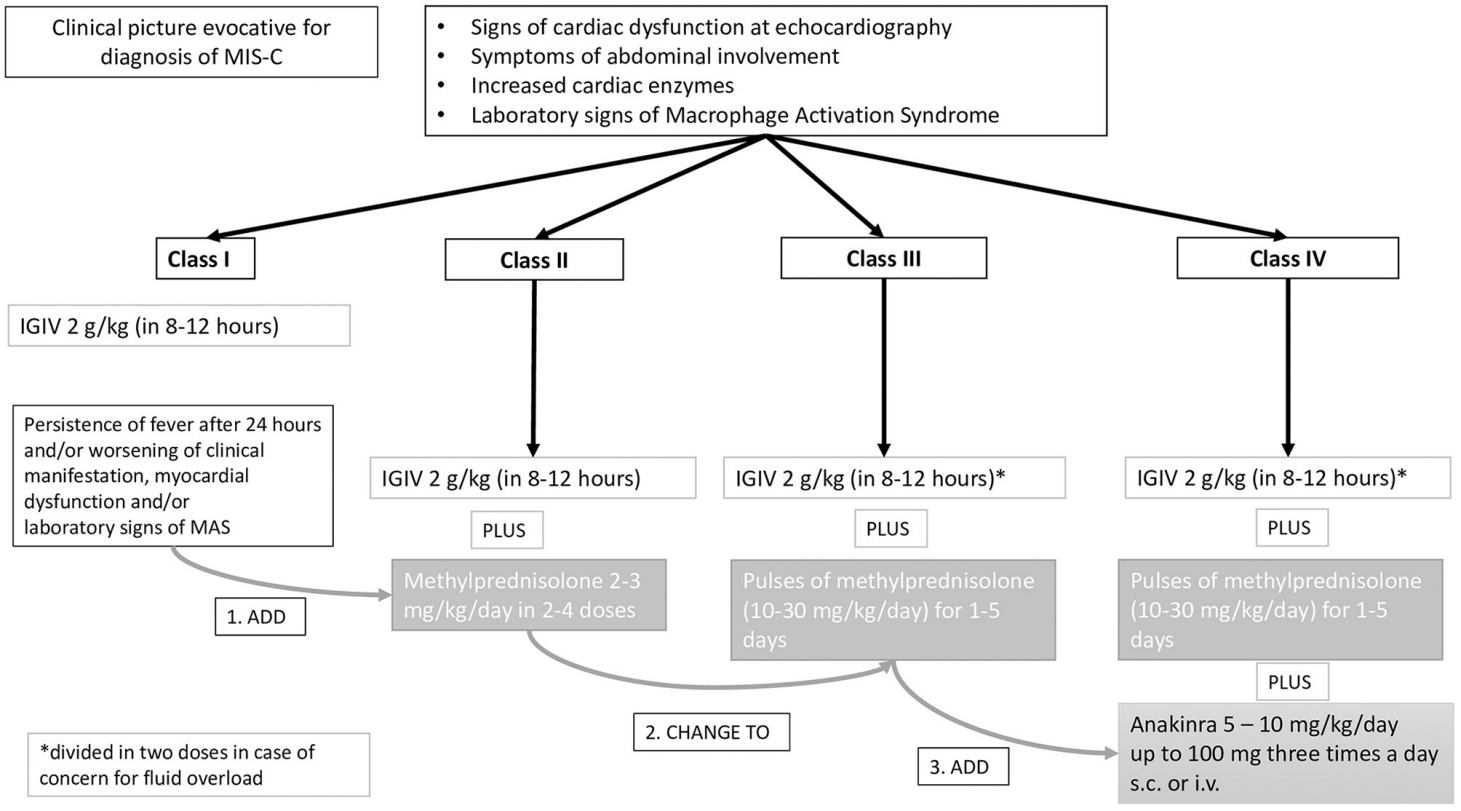
Multistep antinflammatory treatment protocol for MIS-C.

The steps of the protocol, which is shown in Figure 1, were applied as follows. (1) MIS-C patients presenting the American Heart Association (AHA) criteria for complete or incomplete KD (20) with normal left ventricular (LV) ejection fraction (EF>55%) and absence of major abdominal complaints or signs of impending macrophage activation syndrome (MAS) (class I) were given IVIG alone at the standard dosage of 2 gr/kg, as recommended for KD. (2) MIS-C patients with one or more ipokinetic or diskinetic LV segments at the initial echocardiographic evaluation but in the context of a preserved LV global systolic function (EF>50%), and in the absence of major abdominal complaints or signs of impending macrophage activation syndrome (MAS) (class II) were given IVIG plus glucocorticoids upfront by administering intravenous methylprednisolone at 2–3 mg/kg/day in two to four daily doses. (3) MIS-C patients with signs of LV global systolic dysfunction (i.e. EF between 50 and 35%, and/or increased cardiac troponin T level or N-terminal B-type natriuretic peptide level > 1,000/pg/ml) (21), significant abdominal involvement, or impending MAS (class III) were given IVIG plus glucocorticoids as pulses of 10–30 mg/kg/day (maximum 1 gr/day) for 1–5 days (4) MIS-C patients with severe LV global systolic dysfunction (LV EF lower than 35%) and/or arrhythmias (i.e advanced atrioventricular block), hypotension or overt MAS (class IV) were simultaneously started with anakinra at 5–10 mg/kg/day, administered subcutaneously or, in the sickest patients, intravenously.

In patients who did not improve within 24–48 h with the initially selected therapeutic intervention, treatment was escalated throughout the steps outlined above.

For patients with severely depressed cardiac function, we closely monitored for fluid overload, considering adjunctive use of diuretics with IVIG administration possibly given in divided doses (1 g/kg daily over 2 days).

**Figure 2:**
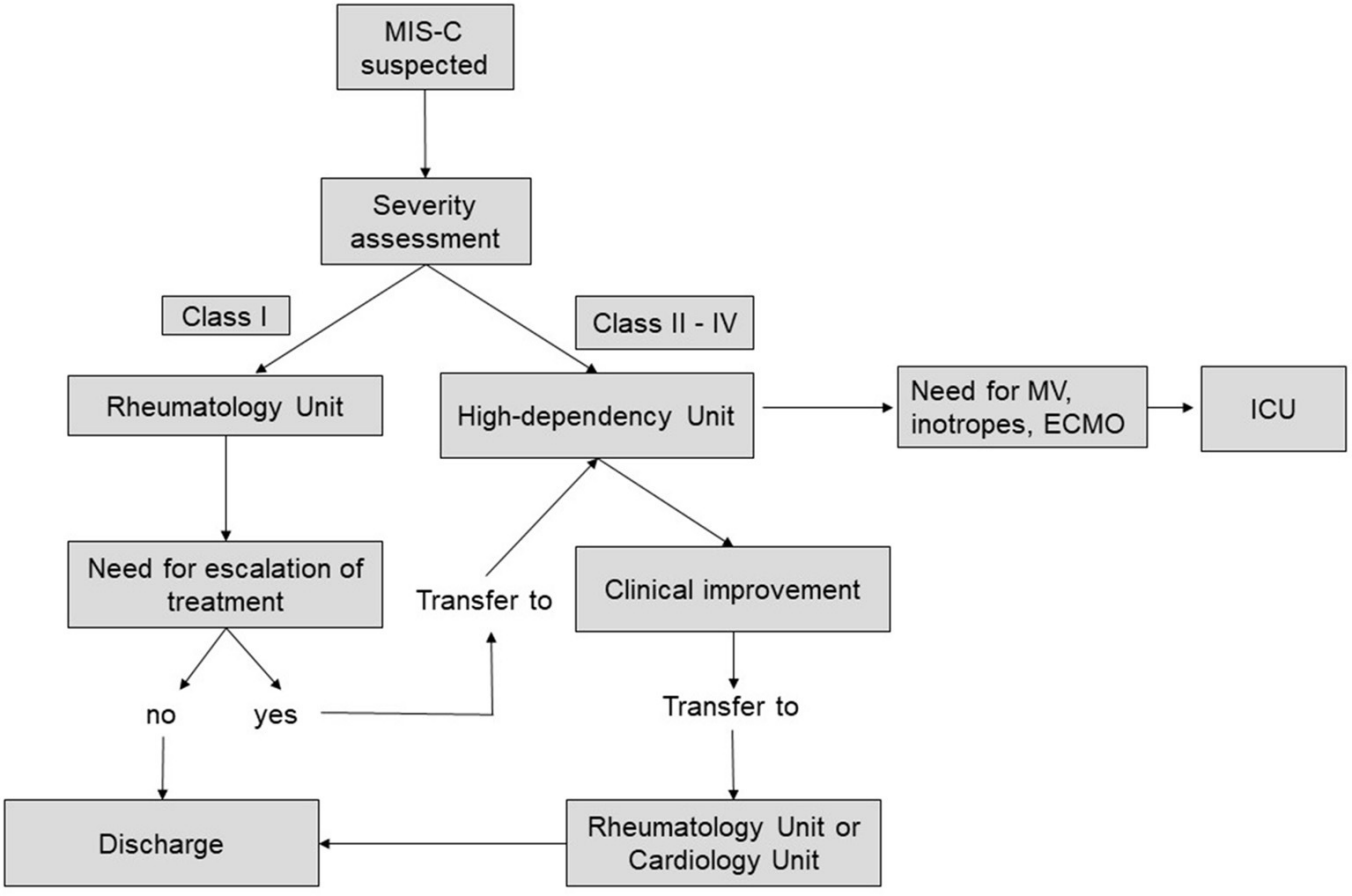
Flow-chart showing location of care for patients with MIS-C.

According to our Institute policy, class I patients were managed in the general ward or in the Rheumatology Unit. Patients assigned to class II-IV or patients in class I with worsening of clinical conditions, were admitted to high-dependency unit until stabilization and then transferred to cardiology unit or rheumatology clinics depending on the characteristics of the cardiac involvement (Figure 2).

## Results

From 1^st^ April 2020 to 1^st^ June 2021 23 patients (11 females, 48, 2%) with MIS-C were treated according to the multistep therapeutic protocol illustrated above. The median age at fever onset was 5, 8 (range 2, 4–12, 3) years and the median time between the onset of fever and hospital admission was three (range 2–4) days. No patients had comorbidities.

In patients with confirmed SARS-CoV-2 infection (i.e. patients who had nasopharyngeal swab positive for SARS-CoV-2), the median time between the infection and MIS-C onset was 4 weeks.

The main clinical and laboratory features of the 23 patients at the time of admission are reported in Table 2. Five and 7 patients met the AHA criteria for complete or incomplete KD, respectively.

**Table 2:** Main clinical features and laboratory studies at presentation of children treated for MIS-C.

**Patient characteristics**	
Median age (range), yr	5, 8 (2, 4–12, 3)
Male, *n* (%)	12 (52, 2%)
Race/Ethnicity	
Asian	1 (4%)
Black/African American	1 (4%)
White	21 (91%)
Hispanic	6 (26%)
Non-Hispanic	15 (65%)
SARS-CoV-2 status	
Nasopharyngeal PCR positive	3 (13%)
Positive serology	19 (83%)
Confirmed COVID-19 exposure	2 (9%)
**Presenting symptoms**	*n (%)*
Fever	23 (100%)
Rash	13 (57 %)
Conjunctivitis	18 (78 %)
Cheilitis	14 (61 %)
Cervical lymphadenopathy	13 (57 %)
Gastrointestinal (abdominal pain, vomiting, and/or diarrhea)	19 (83 %)
Respiratory (dyspnea, cough)	8 (35 %)
Neurological	5 (22 %)
Myalgia/myositis	11 (48%)
**Heart involvement**	20 (87%)
Hypotension	3 (13 %)
Pericarditis	11 (48 %)
Myocarditis	11 (48 %)
Myocardial dysfunction	9 (39 %)
Chest pain	4 (17 %)
**Blood tests**	*median [IQR]*
WBC count (×103/μL)	9, 89 [8, 56, 12, 16]
Neutrophil (×103/μL)	7, 14 [5, 27–9, 52]
Lymphocyte (×103/μL)	1, 43 [0, 91–2, 74]
Platelets (×103/μL)	183, 5 [152–324, 25]
C-reactive protein (mg/dL)	15, 1 [6–18]
Erythrocyte sedimentation rate (mm/1 h)	60 [52.5–67]
Ferritin (*n*g/mL)	382 [211, 5–522]
D-dimer	2, 9 [2, 4–4.7]
NT- pro–BNP (pg/mL)	1,546 [421, 9–4,217]
Troponin T (*n*g/L)	<0, 1 [<0, 1– <0, 1]
Albumin (g/dL)	2, 91 [2,15]
Aspartate transaminase (U/L)	37 [25–45]
Alanine transaminase (U/L)	25 [12–31]

With the application of our severity assessment tool, six patients were assigned to class I, 9 to class II, 5 to class 3, and 3 to class IV.

According to this, in the first 48 h after admission, six patients were given IVIG alone, 14 IVIG plus intravenous methylprednisolone (2–3 mg/kg/day in 9 patients and pulses of 30 mg/kg/day in five patients) and three IVIG, methylprednisolone and anakinra.

**Figure 3:**
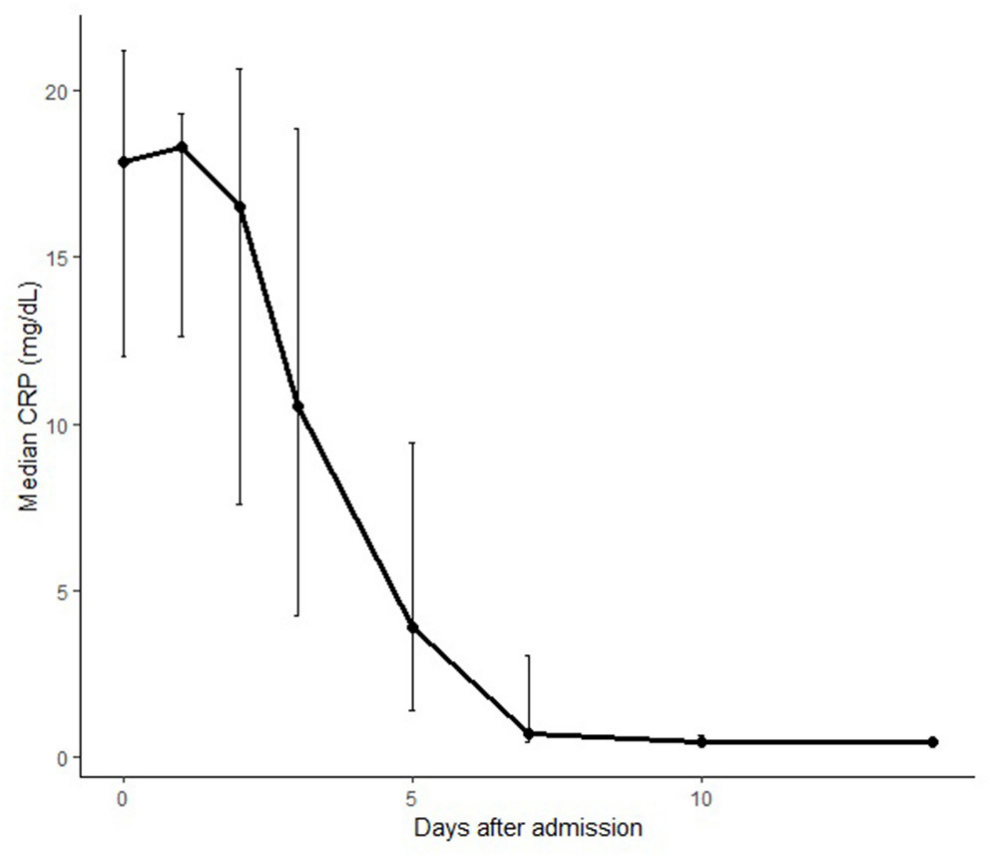
Median C reactive protein levels of patients treated for MIS-C during the disease course.

In 18 (78%) patients, the treatment allocated by the severity score was able to prevent disease progression and to achieve a rapid control of fever, inflammatory markers (Figure 3) and cardiac involvement.

In 5 (22%) patients a subsequent therapeutic escalation was required, due to persistence of fever or worsening of cardiac function. Two patients received methylprednisolone (3 mg/kg/day) after IVIG monotherapy and 3 patients required second-line treatment with anakinra (2–3 mg/kg twice a day IV) for lack of improvement after 3 to 6 days.

In these five patients a full normalization of inflammation markers and cardiac function was rapidly obtained.

Altogether, 23 patients were treated with IVIG, 19 with glucocorticoids, and 6 with anakinra. Patients in severity class I received antiplatelet prophylaxis with acetylsalicylic acid; all remaining patients received low molecular weight heparin prophylaxis.

No patient required admission to the ICU or invasive mechanical ventilation, extracorporeal circulatory and respiratory support and no patients needed administration of inotropic drugs.

The median time of normalization of C reactive protein levels was 9.5 (range 6–16) days (Figure 3).

Five patients were found to have prolonged QTc interval on EKG during hospitalization. Two of them were temporarily treated with beta blockers therapy. Arrhythmia resolved in all patients.

Eleven patients (48%) had coronary involvement: six patients (26%) were found with coronary artery dilations, four patients (17%) with small coronary artery aneurysm and one patient (4%) with medium coronary artery aneurysm.

All cardiac manifestations normalized prior to hospital discharge without developing any sequelae in all but one patient who showed multiple coronary artery aneurysms at last echocardiogram.

All patients were discharged after a median of 20 (range 14, 5–26, 5) days after admission.

The safety of the treatment was good, with only three patients experiencing transient hypertension related to glucocorticoid treatment. No patient had infections related to the immunosuppressive treatment.

## Discussion

We have described our experience with the first 23 patients with MIS-C treated in our Hospital with an early multi-step anti-inflammatory treatment protocol, based on therapeutic interventions tailored based on the severity of the initial clinical manifestations, and rapidly escalated in case of insufficient improvement within the first 24–48 h. Moreover, an integrated multi-specialist organization centered on the presence of a pediatric high-dependency unit closely coordinated with pediatric rheumatologists and cardiologists allowed a similar logistic multi-step approach according to the different disease severity.

With the application of this therapeutic strategy, none of our patients required ICU admission and the outcome was uneventful in all but one patient, a 1-year-old girl assigned to class I on admission, who developed multiple coronary artery aneurysms 2 weeks after receiving treatment with IGIV. The treatment was overall well tolerated, with the exception of the transient occurrence of glucocorticoid-related hypertension in three patients. Although our findings are limited by the small size of our series, they compare favorably with most literature data, which report a significant frequency of ICU admission, need for vasoactive medications and mechanical ventilation, ranging from 60 to 80%, 12 to 47% and 5 to 49% respectively (5, 6, 8, 9, 21, 22).

Of note, in most of our patients, hospital admission and initiation of anti-inflammatory treatment occurred soon after the beginning of symptoms.

Although the lack of fine details about timing of treatment in published case series does not allow us to make comparisons, this observation supports that early identification and prompt initiation of anti-inflammatory therapy may be key factors to obtain a favorable course of the disease and to prevent use of inotropic drugs, mechanical ventilation or ICU admission.

Most of the largest studies available in the literature are multi-centric. It is therefore likely that, beside the different therapeutic strategies, also the different functional organization of each center could have dramatically influenced the results on disease progression and final outcome. In our hospital patients with signs of cardiac involvement or need for close clinical monitoring, were admitted to high-dependency unit until clinical stability was achieved and then transferred to rheumatology or cardiology unit depending on the characteristics of cardiac involvement (Figure 2).

This likely contributed to avoid ICU admissions resulting in significant cost and resource savings and less disruption for patients and their families.

Moreover, since the initial reports of the inflammatory complications related to SARS-COV2 infection, in our hospital, the immuno-rheumatologists raised an internal alert for severe COVID-19 pneumonia (23–25) and, subsequently, for MIS-C (16). Multi-specialist educational meetings with the aim of raising awareness of the disease among emergency and family pediatricians, likely leading to earlier recognition of our cases, were also organized.

On the basis of our experience, considering the wide variability of clinical signs and symptoms at presentation of MIS-C, and the need to induce rapid immunomodulation, we propose a clinical severity stratification tool that allows the clinician to identify the most appropriate anti-inflammatory therapeutic regimen.

So far, the most common therapeutic approach reported in large cohorts of patients with MIS-C, consists in IVIG and systemic glucocorticoids (15).

However, previous studies in adult patients with severe form of COVID-19 pneumonia and evidence of hyperinflammation, have suggested the potential efficacy and safety of the early use of high doses of intravenous anakinra (24, 25), and subsequently confirmed by an extensive metanalysis data (26) and a recent randomized placebo-control study (27).

Moreover, anakinra is becoming increasingly more popular also for the management of KD after failure of IVIG and its efficacy in KD is being scrutinized in a phase IIa trial (28).

More recently, case reports and case series of patients with MIS-C reported the efficacy of treatment with anakinra, highlighting its role particularly in those children who have insufficient response to IVIG and systemic glucocorticoids (29–31).

At the time of admission, patients who meet the case definition criteria for MIS-C are assigned to four severity classes, on the basis of their clinical features, the severity of cardiac involvement expressed by echocardiographic findings and cardiac enzymes levels and the occurrence of blood tests abnormalities suggestive for macrophage activation syndrome, and treated accordingly.

The therapeutic options ranged from IV immunoglobulin alone for patients in class 1 with no sign of cardiac dysfunction to a combination of IVIG plus pulses of methylprednisolone plus anakinra for those in class four with severe cardiac function impairment or signs of macrophage activation syndrome.

We suggest that the treatment allocated by this severity assessment tool may be able to prevent disease progression and to achieve a rapid control of fever, inflammatory markers and cardiac impairment.

Patients who do not respond to initial treatment within the first 48 h enter in the following therapeutic step and receive a more aggressive anti-inflammatory treatment with the aim of rapidly limiting the course of the illness.

Other authors have proposed similar therapeutic algorithms for the management of MIS-C. Schlapbach et al. (32) suggested a disease stratification based on the presence of shock and supported the use of anakinra in severe patients who do not respond to IVIG and methylprednisolone pulses.

Similarly Handerson et al. (15) recommended IVIG and adjunctive low-to moderate dose of glucocorticoids in patients with shock or presenting with concerning features supporting the use of anakinra only for patients with refractory disease.

Differently, we suggest that early identification of more severe patients (i.e. severity class IV of our assessment tool) and initiation of anakinra as first-line therapy may be decisive in turning off the hyperinflammation which underlies the disease and preventing the need for escalation of care.

In our experience only one patient showed residual cardiac lesion at last ultrasound evaluation before hospital discharge, highlighting the potential for medium and long-term complications. Therefore, it is essential to guarantee an adequate follow-up to these patients scheduling both Echocardiograms and EKG at regular intervals for evaluation of ventricular function and coronary artery dimensions after initial diagnosis. Moreover, in patients with a history of ventricular dysfunction, cardiac magnetic resonance imaging (MRI) may be considered 2–6 months after initial diagnosis for evaluation of ventricular function, edema, diffuse fibrosis, and scar (33).

Our findings should be interpreted in the light of some limitations, which include the retrospective nature of the analysis, the relatively low sample size, the absence of a control group and the short-term outcome.

In conclusion, our experience suggests that early aggressive treatment of MIS-C, with therapeutic interventions modulated based on the severity of clinical manifestations and rapidly escalated in case of inadequate therapeutic response may help to prevent the progression of the inflammatory process and to avoid the need of escalation of care and admission to the ICU. The optimal therapeutic approach to MIS-C should be established through multinational consensus initiatives and, whenever possible, randomized controlled trials.

## Data Availability Statement

The raw data supporting the conclusions of this article will be made available by the authors, without undue reservation.

## Ethics Statement

The studies involving human participants were reviewed and approved by Regione Liguria Ethical Board. Written informed consent to participate in this study was provided by the participants' legal guardian/next of kin.

## Author Contributions

GB, RC, and AC conceptualized and designed the study, drafted the initial manuscript, and reviewed and revised the manuscript. DP, GT, and CC collected data, carried out the initial analyses, and reviewed and revised the manuscript. EC, MG, AM, and AR conceptualized and designed the study, coordinated and supervised data collection, and critically reviewed the manuscript for important intellectual content. All authors approved the final manuscript as submitted and agree to be accountable for all aspects of the work.

## Conflict of Interest

The authors declare that the research was conducted in the absence of any commercial or financial relationships that could be construed as a potential conflict of interest.

## Publisher's Note

All claims expressed in this article are solely those of the authors and do not necessarily represent those of their affiliated organizations, or those of the publisher, the editors and the reviewers. Any product that may be evaluated in this article, or claim that may be made by its manufacturer, is not guaranteed or endorsed by the publisher.

## Abbreviations

MIS-C: multisystem inflammatory syndrome in children
KD: Kawasaki disease
ICU: intensive care unit
IVIG: intravenous immunoglobulin
LV: left ventricular
EF: ejection fraction
MAS: macrophage activation syndrome.
